# Effect of four additional physical education lessons on body composition in children aged 8–13 years – a prospective study during two school years

**DOI:** 10.1186/1471-2431-13-170

**Published:** 2013-10-17

**Authors:** Heidi Klakk, Mai Chinapaw, Malene Heidemann, Lars Bo Andersen, Niels Wedderkopp

**Affiliations:** 1 Centre of Research in Childhood Health, Institute of Sports Science and Clinical Biomechanics, University of Southern Denmark, Odense, Denmark; 2 University College Lillebaelt, Odense, Denmark; 3 Department of Public and Occupational Health, EMGO Institute for Health and Care Research, VU University Medical Center, Amsterdam, the Netherlands; 4 Hans Christian Andersen Children’s Hospital, Odense University Hospital, Odense, Denmark; 5 Orthopedic Department, Institute of Regional Health Services Research, University of Southern Denmark, Hospital of Middelfart, Middelfart, Denmark; 6 Department of Sport Medicine, Norwegian School of Sport Sciences, Oslo, Norway; 7 Institute of Sports and Clinical Biomechanics (IOB), Research in Childhood Health (RICH), University of Southern Denmark, Campusvej 55, 5230, Odense M, Denmark

**Keywords:** School-based intervention, BMI, DXA, Total body fat percentage, Children, Obesity prevention, Longitudinal study

## Abstract

**Background:**

Strategies for combating increasing childhood obesity is called for. School settings have been pointed out as potentially effective settings for prevention. The objective of this paper was to evaluate the effect of four additional Physical Education (PE) lessons/week in primary schools on body composition and weight status in children aged 8–13.

**Methods:**

Children attending 2nd to 4th grade (n = 632) in 10 public schools, 6 intervention and 4 control schools, participated in this longitudinal study during 2 school years. Outcome measures: Primary: Body Mass Index (BMI) and Total Body Fat percentage (TBF%) derived from Dual Energy X ray Absorptiometry (DXA). Secondary: the moderating effect of overweight/obesity (OW/OB) and adiposity based on TBF% cut offs for gender.

**Results:**

Intervention effect on BMI and TBF% (BMI: β -0.14, 95% CI: -0.33; 0.04, TBF%: β -0.08, 95% CI:-0.65;0.49) was shown insignificant. However, we found significant beneficial intervention effect on prevalence of OW/OB based on BMI (OR 0.29, 95% CI: 0.11;0.72). The intervention effect on adiposity based on TBF% cut offs was borderline significant (OR 0.64, 95% CI:0. 39; 1.05).

**Conclusion:**

Four additional PE lessons/week at school can significantly improve the prevalence of OW/OB in primary schoolchildren. Mean BMI and TBF% improved in intervention schools, but the difference with controls was not significant. The intervention had a larger effect in children who were OW/OB or adipose at baseline.

## Background

The recent increase in prevalence of childhood overweight (OW) and obesity (OB) has been a growing concern to public health as it is linked to subsequent morbidity and mortality both in adolescence and adulthood [1,2]. Physical activity is essential for the wellbeing and normal growth of children and youth and plays an important role in the prevention of OW and OB and related morbidities [3,4]. Schools are recognized as potentially effective settings for public health initiatives, as they access a large population of children and youth across a variety of ethnic and socioeconomic groups without stigmatizing specific subgroups of high-risk children. The World Health Organization (WHO) specifically identified schools as a target setting for the promotion of physical activity among children and youth [5].

The last decades a considerable number of school-based physical activity promotion and overweight prevention studies have been conducted and their effectiveness on health outcomes evaluated and reviewed [5-7]. The conclusions of these reviews are divergent depending on setting, target group, intervention programs and choice of health outcomes. Despite positive tendencies, intervention effects on overweight and obesity are limited and differ between studies [5-7].

One of the challenges with body composition measures in school studies is that if the majority of school children are normal weight (NW), intervention effects are generally insignificant or very small. In Denmark prevalence of OW and OB in children varies from 12% to 25% depending on age, area and choice of measurement [8-10] and whether obesity is included or not [11].

Body Mass Index (BMI) is the most commonly used measure in studies on OW/OB. It is a weight for height measure and does not take the proportion or distribution of fat mass into account. Though BMI (weight/height^2^) has been shown to strongly correlate with Total Body Fat (TBF) in children [12,13], misclassification of OW and OB is evident. When BMI is compared to more accurate measures of adiposity such as body fat percentage measured by Dual Energy X ray Absorptiometry (DXA) high specificity but low sensitivity for BMI is found [14,15].

There is mounting evidence that PE-based strategies within school studies are effective in increasing physical activity [16,17] and hence may contribute to the prevention of OW and OB. Furthermore, school-based PE interventions are theoretically appealing because adherence with the intervention is potentially high as PE lessons are mandatory. In order to make changes in the school physical education curriculum adaptable and sustainable it is recommended to involve stakeholders (politicians, parents, teachers and children) in the design and provide flexible and easily adaptable solutions. Such policies and solutions could potentially be incorporated and sustained on a population level if shown effective [17]. The CHAMPS study-DK is an evaluation of such a natural experiment where a local community decided to increase the amount of PE lessons in public schools and evaluate the effects on various health outcomes.

This specific study aims to evaluate the effect of 2 years of four additional PE lessons per week at primary schools on body composition and weight status in children aged 8 to 13. The primary outcomes were BMI, TBF% and prevalence of overweight and adiposity. Secondly, the moderating effect of baseline overweight and adiposity was examined.

## Methods

### Design

The CHAMPS study-DK can be described as a quasi experimental study evaluating a natural experiment [18] including 10 public schools – 6 intervention and 4 control schools - in the Municipality of Svendborg (explained in detail elsewhere) [19]. The present study includes baseline and two years follow up data of body composition measures of the pupils attending 2^nd^-4^th^ grade. All children and parents from the 10 participating schools received information about the study through school meetings and written information. Parents signed informed consent forms for joining the project and an additional one for participating in the DXA scans. Participation was at any time voluntary. Permission to conduct The CHAMPS study–DK was granted by the regional scientific ethical committee of Southern Denmark (ID S-20080047).

### Collaboration with the municipality

Initially all 19 primary schools in the municipality of Svendborg, Denmark, were invited to participate in the project as sports (intervention) schools. Ten of the 19 schools agreed to be sports schools, but only six schools were willing to finance the additional lessons. The decision of additional research was made after the 9 schools had resigned from being a sports school. The municipality was asked to provide six matched control schools but only four schools agreed to become a control school. The six intervention schools and the four control schools were matched based on school size, urban-suburban/rural area and socio-economic position.

Though it is the capital of the municipality, Svendborg is a small town with surrounding rural districts. The 10 participating schools represent half of the public schools in the municipality. Four schools were urban/suburban (two intervention/two control) and six were rural (four intervention/two control). Of the non-participating nine schools, six were urban/suburban and three were rural schools.

Parents and children were unaware of the initiation of this project until two months before the following school year avoiding parents making an influenced school choice [19].

### The school- based PE program

The school leaders and PE teachers of the intervention schools were invited to design the set-up for an optimal PE intervention. The number of children per PE teacher was on average 20, and girls and boys had PE together. The six intervention schools chose to implement four additional PE lessons per week to their usual PE program (resulting in a minimum of 4.5 hours PE per week divided over at least 3 sessions of at least 60 minutes) and to educate the specialized PE-teachers in specific age-related training principles. The four control schools continued their regular PE curriculum (i.e. 2 PE lessons/week resulting in 1.5 hours/week) [19].

### Participants and measurements

All children attending 2^nd^ to 4^th^ grade in 2008 were invited for a DXA scan. DXA scans, height, weight and pubertal stage were assessed according to a standardized procedure at the same day and location. Only children with complete data at both time points were included in the analysis.

### Body Mass Index (BMI)

Weight was measured to the nearest 0.1 kg on an electronic scale, (Tanita BWB-800S, Tanita Corporation, Tokyo, Japan) wearing light clothes. Height was measured to the nearest 0.5 cm using a portable stadiometer, (SECA 214, Seca Corporation, Hanover, MD). Both anthropometrics were conducted barefoot. BMI was calculated as [weight (kg)/height^2^ (m)].

### Overweight/obesity

BMI classifications for normal weight (NW), OW, and OB were defined using age- and sex specific cut-offs as recommended by the International Obesity Taskforce recommendations [20]. Dichotomized categories were made for weight classes NW as one and OW/OB in another category to easier compare with the dichotomous variable of normal fat /adipose as described beneath according to Williams [21].

### Total Body Fat Percentage (TBF%)

Total body fat mass was measured by Dual Energy X ray Absorptiometry (DXA), (GE Lunar Prodigy, GE Medical Systems, Madison, WI), ENCORE software (version 12.3, Prodigy; Lunar Corp, Madison, WI). The procedure took place at Hans Christian Andersen Children’s Hospital, Odense University Hospital, Denmark. The child was instructed to lie still in a supine position wearing underwear, a thin T-shirt, stockings and a blanket for the duration of the x-ray. All scans were performed by two different operators and analyzed by one on them. The DXA machine was reset every day, following standardized procedures. TBF% was calculated for each participant from the equation: [(TBF (g) x 100)/ weight (g)].

### Adiposity

Cut-offs to classify children as normal-fat or adipose were defined using the cardiovascular health- and gender-related TBF% standards according to Williams *et al *[21]. These standards were derived from a cross sectional study on 3320 children and adolescents aged 5 to 18 years. Equations developed specifically for children using the sum of subscapular and triceps skinfolds were used to estimate percentage fat. Body density was estimated from age and the sum of triceps and subscapular skinfolds and was subsequently used to derive total percentage body fat. Their analysis resulted in recommended health related cut-offs for adiposity for boys at ≥ 25% TBF and ≥ 30% TBF for girls [21].

### Pubertal stage

Puberty was defined by self-assessment. The Tanner pubertal stages self-assessment questionnaire (SAQ) used in this study consists of drawings of the 5 Tanner stages [22]. Boys were presented with pictures and text of Tanner staging for pubic hair development, whereas girls were presented with pictures and text representing breast development and pubic hair [23]. Explanatory text in Danish supported the self-assessment. The children were asked to indicate which stage best referred to their own pubertal stage. The procedure took place in a private space with sufficient time to self assess the pubertal stage.

### Statistical analysis

Summary statistics were calculated (means and SD) for the descriptive part on anthropometrics. Differences in OW/OB and adiposity prevalence were tested using Chi square tests. Fisher exact was used for testing differences in pubertal status between schools. Significance level was set at p ≤ 0.05.

To estimate the effect of school type multivariate multilevel mixed effect regression analysis using hierarchical models were used based on the intention to treat principle. Individual, class and school were considered random effects. Analyses were adjusted for age, gender and puberty (and height when TBF% was the outcome variable). Effect modification by gender, age and baseline OW/OB and adiposity category was explored by adding an interaction term between the moderator and school type (intervention versus control). If the interaction term was significant (p < 0.10), subgroup analyses were performed.

In a sensitivity analyses we compared the effect of the intervention based on the non-imputed sample with a sample with imputed data. We imputed missing information on covariates and outcomes (n = 22 to n = 84) using chained equations ("mi impute chained" in STATA) [24]. All covariates, the respective outcomes, and the cluster variables school and class were included in the imputation approach. Beta coefficients and SEs were based on 20 imputed datasets.

## Results

**Figure 1:**
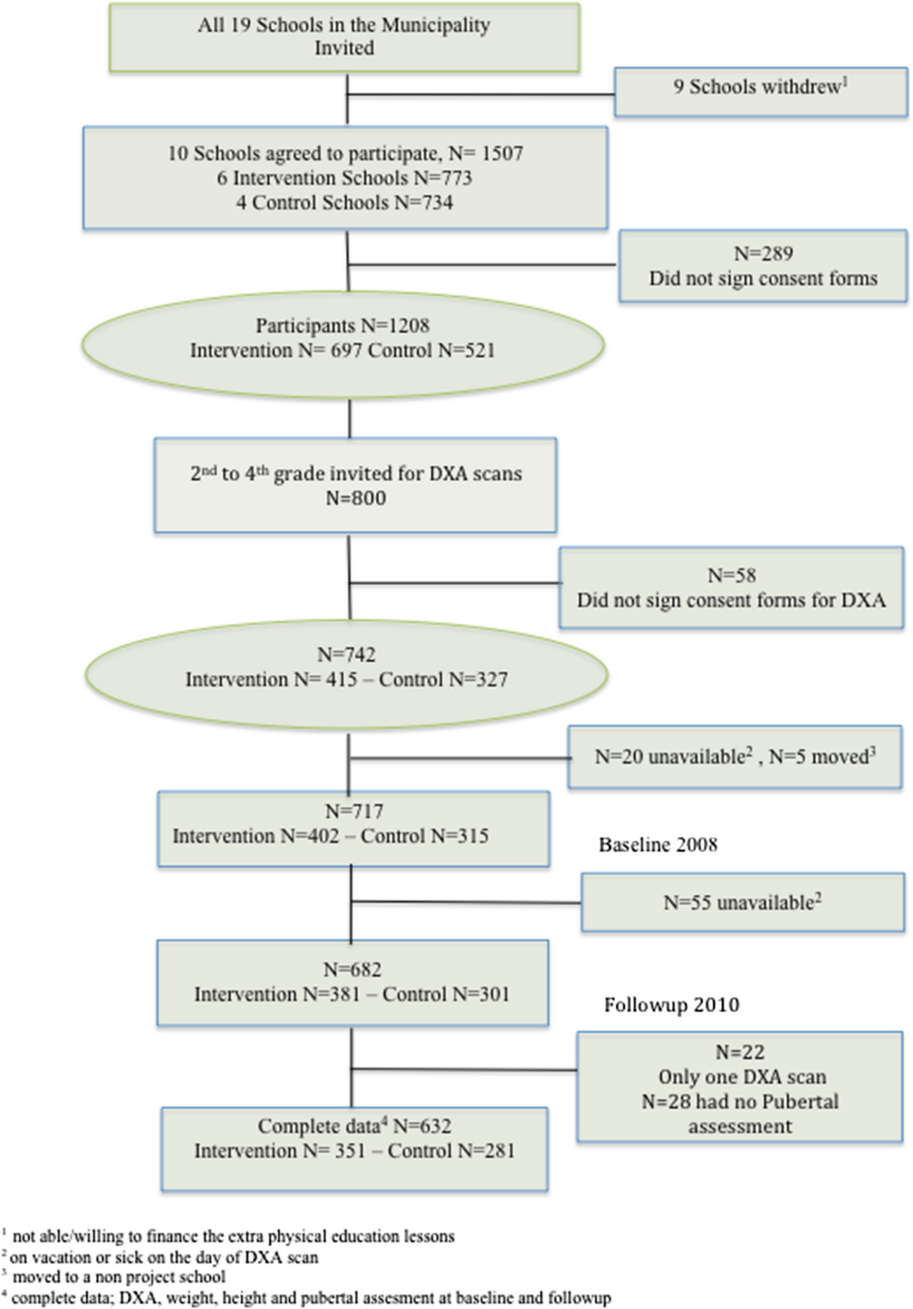
Flowchart of Participants of the DXA, anthropometry and pubertal assessment.

In the overall study, 1507 children from the preschool year to 4^th^ grade (age range 5.5-12 years) were invited to participate in The CHAMPS study-DK from baseline in September 2008, of which 1218 (80%) accepted. All 800 children attending 2^nd^ to 4^th^ grade (7.7-12.0 years) at baseline were invited for a DXA scan. In total 742 children (93%) accepted the invitation of these 739 (99.6%) children had a DXA scan at one time point, 717 children (97%) had a DXA scan at baseline and 682 (92%) at follow-up, 660 children (89%) had measurements at both time points, but when adjusting regressions for the chosen covariates this number was reduced to n = 632 (86%) (Figure 1). We found no significant differences between intervention or control schools regarding age, gender, anthropometry, prevalence of OW/OB, adiposity and pubertal stages at baseline. Boys and girls at all ages were equally represented in the sample (Table 1). Individuals with missing data or lost to follow up (n = between 85 and 107) had higher mean values of height, weight, BMI, TBF% and higher prevalence of OW/OB by BMI and TBF. School type was equally represented in children with missing data/lost to follow up (63% intervention, 47% control schools).”

**Table 1:** Baseline values for key variables by school type and gender

	**Intervention schools**			**Control schools**		
	**Girls**	**Boys**	**All**	**Girls**	**Boys**	**All**
	**54%**	**46%**		**47%**	**53%**	**N = 281**
	**N = 191**	**N = 160**	**N = 351**	**N = 133**	**N = 148**	
Key variables	Baseline values Mean (SD)					
Age	9.2 (0.9)	9.3 (0.8)	9.3 (0.9)	9.4 (0.9)	9.3 (1.0)	9.4 (0.9)
Height	138.3 (7.3)	140.4 (7.3)	139.2 (7.3)	138.5 (7.1)	139.7 (8.3)	139.1 (7.8)
Weight	32.2 (6.0)	32.9 (6.3)	32.5 (6.1)	32.4 (6.5)	32.8 (6.5)	32.6 (6.5)
BMI	16.7 (2.1)	16.6 (2.2)	16.7 (2.2)	16.8 (2.2)	16.7 (2.0)	16.7 (2.1)
TBF%	22.6 (7.3)	17.1 (7.6)	20.1 (7.9)	23.7 (7.6)	17.5 (7.3)	20.5 (8.1)
Prevalence:%(n):						
OW/OB (BMI)	13 (25)	8 (13)	11 (38)	11 (15)	10 (14)	10 (29)
Adiposity (TBF%)	16 (31)	14 (23)	15 (54)	23 (31)	18 (26)	20 (57)
Puberty	N = 189	N = 157	N = 347	N = 131	N = 148	N = 280
1	74 (140)	48 (75)	62(216)	79 (104)	56 (82)	67 (187)
2	23 (43)	47 (75)	34(118)	20 (26)	41 (61)	31 (87)
3	2 (4)	4 (7)	3 (11)	1 (1)	3 (5)	2 (6)
4	1 (2)	0	1 (2)	0	0	0
5 (none)	-	-	-	-	-	-

### Primary outcome

Multilevel linear regression analysis showed no significant intervention effect on BMI or TBF% (BMI: β -0.14, 95% CI: -0.33; 0.04; TBF%: β -0.08, 95% CI:-0. 65;0.49). Sensitivity analysis comparing the intervention based on the non-imputed sample (n = 632) with the sample with imputed data (n = 739) did not change effect estimates significantly (BMI: β -0.14, 95% CI: -0.45; 0.07; TBF%: β -0.12, 95% CI:-0.73;0.49).

The intervention had a significant beneficial effect on OW/OB prevalence. Children at intervention schools had a significant reduced risk of becoming OW/OB (OR 0.29, 95% CI: 0.11;0.72, p = 0.01) after 2 school years compared to children at control schools. The intervention effect on prevalence of adiposity was smaller and borderline significant (OR 0.64, 95% CI:0.39; 1.05, p = 0.08) (Table 2).

**Table 2:** Crude means/prevalence and adjusted effect size by school type

	**Baseline**	**Follow up**	**Effect (95% CI)**	**ICC**
	**N = 632**	**N = 632**		
**BMI**	**Mean(SD)**	**Mean(SD)**	**β**	**School/class**
Intervention	16.7 (2.2)	17.7 (2.5)	-0.14 (-0.33; 0.04) ^1^	0.02/0.02
Control	16.8 (2.1)	17.9 (2.6)		
TBF%				
Intervention	20.1 (7.9)	21.8 (7.6)	-0.08 (-0.65; 0.49) ^2^	
Control	20.5 (8.1)	22.1 (8.6)		
** Prevalence%(n)**			** OR**	
OW/OB				
Intervention	11 (38)	9 (30)	0.29 (0.11; 0.72) ^3^	<0.01/<0.01
Control	10 (29)	13 (37)		
Adiposity				
Intervention	15 (54)	19 (68)	0.64 (0.39; 1.05) ^3^	<0.01/<0.0001
Control	20 (57)	27 (76)		

^1^ adjusted for baseline BMI, gender, age and puberty.

^2^ adjusted for baseline TBF%, gender, age, puberty and height.

^3^ adjusted for being OW/OB (adipose by TBF%) at baseline, gender, age and puberty.

### Secondary outcome

There was no significant effect modification by age or gender. There was however, a significant moderating effect of OW/OB (β -.48, p = 0.07) on BMI. Additionally a significant moderating effect of adiposity β -.14 (p = 0.05) on TBF% was found. Therefore subgroup analyses were performed in OW/OB (n = 67) versus NW (n = 565) children and adipose (n = 111) versus normal fat (n = 521) children. The intervention effect on BMI in OW/OB children was larger but not significant (β -0.5, 95% CI: -1.6; 0.6) compared to NW children (β -0.09, 95% CI: -0.24; 0.06). The intervention effect on TBF% was even larger although not significant in adipose children (β -1.18. 95% CI: -2.6; 0.2) compared to normal fat children (β 0.16, 95% CI: -0.4; 0.74).

## Discussion

This paper aimed to evaluate the effect of 2 years of four additional PE lessons per week on body composition and weight status of primary school children aged 8 to 13 yrs. Four additional PE lessons at school had a significant beneficial effect on the prevalence of OW/OB. Moreover, weight status was a significant effect modifier with a larger effect in OW/OB and adipose children.

Our findings support the results of other recently published school studies on additional PE lessons [16,25-27]. We found no significant effect on BMI although the effect size (β -0.14) on BMI in our study was comparable with those calculated in a meta-analysis (β -0.15; β - 0.20) evaluating the effect of school-based interventions (including PA, dietary and family based programs) on BMI [28,29] suggesting that our study was underpowered. Post hoc power analysis showed that given the sample size and the observed effect size in this study power was 0.42. This raises the question whether the effect size is “clinically irrelevant” or the sample size too small to reach significance for relevant but small changes. It is beyond the scope of this paper to answer that question, but some evidence is given that any stabilization of BMI might be preventive of future co-morbidity in the adult population [30]. It could be speculated that BMI is an inappropriate measure of changing body composition in physical activity intervention studies, as a consequence of the intervention could theoretically be an increase in lean mass. Based on the DXA scans this was not the case in this study: we found no significant intervention effect on lean mass.

We have not identified any recent school-based intervention studies including DXA scan for the assessment of total body fat percentage. Kriemler *et al* (KISS) reported a significant effect on total body fat measured by a z-score on sum of four skinfolds (β -0.12 95% CI: -0.21—0.03) [31]. Their intervention was comparable in volume to the CHAMPS study-DK (5 lessons a week and additional PA homework for 1 year). However prevalence of OW/OB was higher in the KISS population compared to CHAMPS study-DK (26% versus 11%). This higher starting point leaves more room for improvement in both total body fat as well as the prevalence of OW/OB. This is in accordance with the higher effect size we found in OW/OB children compared NW children. Gonzalez-Suarez *et al *[32] also reported a significant intervention effect in a meta-analysis including 6 of 19 school based intervention studies on percentage body fat based on skinfolds. Although sum of skinfolds have been shown to highly correlate with DXA assessment of body composition in children [33] estimations from skinfolds must still be considered as a less direct and precise measurement of adiposity than DXA. Our results might be negatively biased, as the group comprising children with missing data was more OW/OB than those in analysis. Imputing missing data did not change effect estimations significantly, but increased the effect estimates on change in TBF% in favour of the intervention schools.

Few school-based physical activity promotion studies report the effect on overweight prevalence or effect modification of overweight. One study by Marcus *et al *[34] measured the effectiveness of a school-based intervention on prevalence of OW/OB and found in agreement with our results, that the intervention significantly reduced the prevalence at intervention schools. Graf *et al *[27] on the other hand found that neither prevalence nor incidence differed between intervention and control schools after 4 years of intervention. This might be explained by a “levelling off” of intervention compliance during the four years, as reported by the researchers. Also Sollerhed *et al *[16] reported no difference in incidence of OW/OB between intervention and control schools during one school year. Intervention being 4 PE lessons per week compared to the mandatory 1–2 PE lessons per week. The study by Sollerhed *et al* might be too small to detect changes in incidence of OW/OB (defined by BMI) in a relatively healthy and lean population (132 children in two schools in Sweden). Results from a meta-analysis by Gonzalez-Suarez *et al* support our findings on effectiveness on prevalence of OW/OB. The meta-analysis included 19 school-based intervention studies, and besides a significant intervention effect on OW/OB they found that the longer the intervention lasted the larger the effect [32].

### Strengths and limitations

A strength of this study is the real life setting. Given the nature of a natural experiment, the researchers had no influence or control of the content and intensity of the PE lessons besides the anticipation, that the teachers followed the age-related concept as taught to them in workshops during the first school year. As it is a natural experiment results are not dependent on researchers or experts set up of intervention and therefore considered directly transferable into the daily praxis in other school settings. This is of great importance for public health as the intervention is implementable in “the real world” and at relatively low costs. The municipality of Svendborg has subsequently sustained the four additional PE lessons and even expanded this to more grades (kindergarten to 7^th^ grade). In this way the health promotion and prevention is a natural part of the public schools and as stated by several reviews and meta-analysis long lasting and “compulsory” efforts are more likely to be effective [6,35-37]. Also the research part continues and this gives the research team an exquisite opportunity of monitoring the maintenance of effects in the long term.

The fact that additional PE lessons had a positive, though not always significant, effect on all body composition measures and a larger effect in the OW/OB and adipose subgroup, confirms that interventions are more effective if “compulsory” compared to “voluntary”, as suggested by Connelly *et al *[35]. In the CHAMPS study-DK the intervention consisted of four additional mandatory PE lessons in addition to the mandatory two. None of the intervention schools reported any troubles fulfilling that. Other strengths of this study are the large cohort, high participation rate, the objective accurate measurement of body composition and the long duration of intervention without levelling off in compliance.

A limitation to this study is the study sample being “too healthy” with few “at risk” children. This limits generalizability to other countries with higher prevalence of OW/OB and adiposity. Although parental demographic profile of participants resembles that of the target population generalizability of results may not extend beyond this type of population (small town with surrounding rural district in Denmark).”

Another limitation might be the use of TBF% cut offs recommended by Williams *et al *[21]. Williams’ study was established in 1992 and their adiposity measure was, as described in the method section, calculated from skinfolds. These health related cut off points might not be directly transferable to those derived from DXA scans in our study. But we did not, in the literature, find any DXA derived cut off points for children related to biological endpoints. This issue was important, as we wanted a measure of adiposity that is health related.

The design of our study gives certain limitations. Firstly intervention and control schools were matched, not randomized, which may not eliminate the risk of confounding. Secondly we do not know much about the quality of the PE lessons as the schools solely conducted them.

## Conclusion

Four additional PE lessons per week at school did not significantly improve BMI or TBF% at intervention schools compared to control schools, but did significantly improve the prevalence of OW/OB in primary schoolchildren. Our findings support, what has been stated by Harris *et al*[6], that effects of school based preventive interventions on body composition are likely to be insignificant and/or small unless the effect is measured in high risk subgroups, as the majority of the children are normal weight and not expected to change in BMI or TBF%.

Further research on school based interventions should focus on mediation analysis to provide knowledge about what “actually” works and how. Further research is also needed on the relation of body fatness derived from DXA scans and biological endpoints, as reducing the degree or prevalence of adiposity should be linked to health gains and not merely a change of distribution in the population.

## Abbreviations

OW: Overweight; OB: Obesity; DXA: Dual Energy X ray Absorptiometry; TBF%: Total body fat percentage; BMI: Body mass index; PE: Physical Education.

## Competing interests

The authors declare that they have no competing interest.

## Authors’ contributions

HK had full access to all of the data in the study and takes responsibility for the integrity of the data and the accuracy of the data analysis. All authors contributed to important parts of the process: Study concept and design: HK, NW, LBA . Acquisition of data: HK, MSH. Analysis and interpretation of data: HK, MC, NW, LBA. Drafting of the manuscript: HK, MC. Critical revision of the manuscript for important intellectual content: HK, MC, NW, MSH, LBA. Statistical analysis: HK, MC, NW. Obtained funding: NW, LBA. All authors read and approved the final manuscript.

## Pre-publication history

The pre-publication history for this paper can be accessed here:

http://www.biomedcentral.com/1471-2431/13/170/prepub
